# Maternal caffeine intake during pregnancy is associated with birth weight but not with gestational length: results from a large prospective observational cohort study

**DOI:** 10.1186/1741-7015-11-42

**Published:** 2013-02-19

**Authors:** Verena Sengpiel, Elisabeth Elind, Jonas Bacelis, Staffan Nilsson, Jakob Grove, Ronny Myhre, Margaretha Haugen, Helle Margrete Meltzer, Jan Alexander, Bo Jacobsson, Anne-Lise Brantsæter

**Affiliations:** 1 Department of Obstetrics and Gynaecology, Sahlgrenska Academy, Sahlgrenska University Hospital/Östra, SE-416 85 Gothenburg, Sweden; 2 Norwegian Institute of Public Health, Department of Exposure and Risk Assessment, Division of Environmental Medicine, PO Box 4404 Nydalen, NO-0403 Oslo, Norway; 3 Mathematical Sciences, Chalmers University of Technology, SE-412 96 Gothenburg, Sweden; 4 Department of Biomedicine, Aarhus University, Wilhelm Meyers Allé 4, DK-8000 Aarhus C, Denmark, and Bioinformatics Research Centre (BiRC), Aarhus University, CF Møllers Allé 8, DK-8000 Aarhus C, Denmark; 5 Norwegian Institute of Public Health, Department of Genes and Environment, Division of Epidemiology, PO Box 4404 Nydalen, NO-0403 Oslo, Norway; 6 Norwegian Institute of Public Health, Office of the Director-General, PO Box 4404 Nydalen, NO-0403 Oslo, Norway

**Keywords:** preterm delivery, gestational length, small for gestational age, birth weight, growth curve, intrauterine growth restriction, caffeine, coffee, tea, soft drinks

## Abstract

**Background:**

Pregnant women consume caffeine daily. The aim of this study was to examine the association between maternal caffeine intake from different sources and (a) gestational length, particularly the risk for spontaneous preterm delivery (PTD), and (b) birth weight (BW) and the baby being small for gestational age (SGA).

**Methods:**

This study is based on the Norwegian Mother and Child Cohort Study conducted by the Norwegian Institute of Public Health. A total of 59,123 women with uncomplicated pregnancies giving birth to a live singleton were identified. Caffeine intake from different sources was self-reported at gestational weeks 17, 22 and 30. Spontaneous PTD was defined as spontaneous onset of delivery between 22^+0 ^and 36^+6 ^weeks (n = 1,451). As there is no consensus, SGA was defined according to ultrasound-based (Marsal, n = 856), population-based (Skjaerven, n = 4,503) and customized (Gardosi, n = 4,733) growth curves.

**Results:**

The main caffeine source was coffee, but tea and chocolate were the main sources in women with low caffeine intake. Median pre-pregnancy caffeine intake was 126 mg/day (IQR 40 to 254), 44 mg/day (13 to 104) at gestational week 17 and 62 mg/day (21 to 130) at gestational week 30. Coffee caffeine, but not caffeine from other sources, was associated with prolonged gestation (8 h/100 mg/day, *P *<10^-7^). Neither total nor coffee caffeine was associated with spontaneous PTD risk. Caffeine intake from different sources, measured repeatedly during pregnancy, was associated with lower BW (Marsal-28 g, Skjaerven-25 g, Gardosi-21 g per 100 mg/day additional total caffeine for a baby with expected BW 3,600 g, *P *<10^-25^). Caffeine intake of 200 to 300 mg/day increased the odds for SGA (OR Marsal 1.62, Skjaerven 1.44, Gardosi 1.27, *P *<0.05), compared to 0 to 50 mg/day.

**Conclusions:**

Coffee, but not caffeine, consumption was associated with marginally increased gestational length but not with spontaneous PTD risk. Caffeine intake was consistently associated with decreased BW and increased odds of SGA. The association was strengthened by concordant results for caffeine sources, time of survey and different SGA definitions. This might have clinical implications as even caffeine consumption below the recommended maximum (200 mg/day in the Nordic countries and USA, 300 mg/day according to the World Health Organization (WHO)) was associated with increased risk for SGA.

## Background

There is increasing epidemiological evidence that maternal nutrition influences the course of pregnancy as well as fetal growth and development and the risk of disease later in life [1-3]. Maternal diet should ideally supply all vital nutrients but does also, irrespective of composition, contribute contaminants and compounds with pharmacological activity that may have adverse effects. Caffeine is a xanthine alkaloid found primarily in coffee, tea, cocoa, energy drinks and some soft drinks, and is thus consumed on a daily basis all over the world. Caffeine passes the placental barrier freely; the fetus does not express the main enzymes that inactivate it [4,5], and caffeine metabolites have been found to accumulate in the fetal brain [6,7]. In 2005, a Scandinavian expert committee concluded that high caffeine intake may harm the fetus [5]. The current World Health Organization (WHO) guidelines recommend a caffeine intake below 300 mg/day during pregnancy [8], while the American College of Obstetricians and Gynecologists and the Norwegian Food Safety Authority concur with the Nordic Nutrition Recommendations (NNR), recommending a maximum caffeine intake of 200 mg/day [9-11].

Human studies on adverse effects of caffeine have investigated spontaneous abortion, preterm delivery (PTD), fetal death, congenital malformations and fetal growth restriction, with conflicting results for all outcomes [12-23]. PTD and small for gestational age (SGA) at birth are the pregnancy outcomes accounting for most of all neonatal mortality, as well as short-term and long-term morbidity [24-27]. Both are common, complex conditions; the respective prevalences in the Norwegian population are around 7% and 5% [28]. Despite these prevalences, the complexity makes it difficult to measure the effect of a single environmental factor, except in large studies. While some studies have found a higher risk for PTD [29] or early PTD [30] with increasing caffeine intake, most studies on caffeine intake have found no significant association with gestational length as summarized in the meta-analysis by Maslova *et al.*[31] and in the comprehensive review by Peck *et al.*[20]. Although PTD is a heterogeneous pregnancy outcome with different etiologies (for example, for early versus late PTD or for iatrogenic versus spontaneous PTD [32]) it has mostly been studied as one entity, which may obscure associations with subtypes of PTD.

Approximately half of all studies report an adverse effect of caffeine intake on BW, while others have not found any significant associations. Comparability among these studies is problematic due to the use of different standard growth curves or to incomplete or inaccurate assessment of caffeine exposure [5,20]. Peck *et al.* concluded in their review that the evidence for an association between caffeine intake and reproductive health and fetal development is limited by measurement errors as well as by the impossibility of ruling out confounding by pregnancy symptoms such as nausea or environmental factors such as smoking. Caffeine consumption is strongly correlated with smoking, which is known to increase the risk for both PTD and SGA. As mentioned above, there are methodological challenges in the assessment of caffeine intake, both from coffee and other sources. This also applies to preparation and processing, which may change the caffeine content of a beverage considerably. Pregnancy is a time of rapid development and differentiation, therefore there might be a certain time window for a caffeine effect; repeated measurements during pregnancy may thus be desirable [20].

In summary, caffeine is consumed daily by many pregnant women, spontaneous PTD and SGA incur high medical and economic costs and studies on associations between caffeine and pregnancy outcomes are contradictory due to a number of challenges in study design. The Norwegian Mother and Child Cohort Study (MoBa) can meet many of these challenges: with about 108,000 included pregnancies, common complex pregnancy outcomes like PTD and SGA can be studied. With detailed reporting of caffeine intake from various sources and different coffee preparations, assessed at three different timepoints during pregnancy, as well as comprehensive information on lifestyle habits, health and socioeconomic status, MoBa provides a unique chance to study the association between caffeine intake and pregnancy outcomes. By taking caffeine intake from different sources into account, it might be possible to separate caffeine effects from other effects related to the respective sources.

The aim of the present study therefore was to examine the association between maternal caffeine intake from different sources and (a) gestational length, particularly the risk for spontaneous PTD with a subanalysis of early and late spontaneous PTD, and (b) BW and the risk for SGA.

## Methods

### Study population

The dataset is part of the MoBa cohort, initiated by and maintained at the Norwegian Institute of Public Health [28]. In brief, MoBa is a nationwide pregnancy cohort, including more than 108,000 pregnancies during the period 1999 to 2009. Women were recruited by postal invitation in connection with the routine ultrasound examination offered to all pregnant women in Norway at around 17 gestational weeks. Overall, 38.5% of invited women have participated. Participants were asked to fill in questionnaires focused on general health status, lifestyle behavior and diet at gestational weeks 15 to 17 (Q1) and 30 (Q3). At gestational week 22, they completed a food frequency questionnaire (FFQ). All questionnaires are available on the Norwegian Institute of Public Health homepage [33]. This study used data from version 5 of the quality-assured data files made available for research in 2010. Pregnancy and birth records from the Medical Birth Registry of Norway (MBRN) are linked to the MoBa database [34]. Informed written consent was obtained from each participant. The Regional Committee for Medical Research and the Norwegian Data Inspectorate approved the study.

Of the 106,707 pregnancies included in MoBa version 5, 103,835 women gave birth to live-born singletons; 81,301 of these women had answered all three questionnaires. After exclusion of the following medical and pregnancy-related conditions 70,105 pregnancies remained in the study: diabetes mellitus, hypertension, autoimmune disease, inflammatory bowel disease, systemic lupus erythematosus, rheumatoid arthritis, scleroderma, other immune-compromised conditions, *in vitro *fertilization, pre-eclampsia, hypertension, gestational diabetes, placental abruption, placenta previa, cervical cerclage and serious fetal malformations. Women reporting improbable energy intake, that is, <4.5 MJ or >20 MJ, were excluded [35], leaving 69,045 pregnancies. If a woman participated with more than one pregnancy, only the first pregnancy was included, leaving 60,496. Finally, 59,123 women had complete data on pre-pregnancy weight and height.

### Outcome

Gestational age in days was determined by second-trimester ultrasound in 98.3% of pregnancies and based on the last menstrual period in the remaining cases. The expected effect of caffeine on gestational length is minor; results are thus presented in hours instead of days. Spontaneous PTD was defined as birth after preterm labor or prelabor rupture of the membranes between 22^+0 ^to 36^+6 ^weeks, while controls delivered spontaneously between 39^+0 ^to 40^+6 ^weeks. A subanalysis was conducted for the subgroups early (22^+0 ^to 33^+6 ^weeks) and late (34^+0 ^to 36^+6 ^weeks) spontaneous PTD.

BW in grams was registered in the MBRN. As there is still no consensus on standard growth curves, data were analyzed according to three standards based on Northern European populations: ultrasound-based growth curves according to Marsal [36], population-based growth curves according to Skjaerven [37] and customized growth curves according to Gardosi [38].

The difference between BW and expected BW was calculated as a percentage of expected BW. This implicates that gestational length is taken into account by the definition of our outcome variable and analysis were not adjusted for gestational age. Percentage was used instead of the difference in g, as a slight weight difference matters much more if the expected BW for a preterm infant is very low compared with a normal-weight infant born at term. While the original outcome of the linear regression thus was a percentage of the expected BW, we chose to present the results in g for babies with an expected BW of 3,600 g, the rounded-out median BW in our study population (actual median: 3,620 g). SGA was defined according to the above-mentioned authors' respective definitions: less than-2 SD (Marsal) or <tenth percentile (Skjaerven, Gardosi). Standard deviation and percentiles used are based on the reference population in these publications, not on our study population, which is a highly selected subpopulation of the MoBa population, as described above.

### Caffeine intake

The MoBa FFQ is a semiquantitative questionnaire designed to record dietary habits and intake of dietary supplements during the first four to five months of pregnancy. Women reported their beverage consumption in cups per day, week or month. Coffee was specified as either filtered, instant, boiled/pressed, decaffeinated, caffè latte/cappuccino, espresso or fig/barley coffee. One cup was defined as 125 ml. In the case of black tea, one cup was defined as 250 ml. One glass of sugar-sweetened or diet cola, energy drink or chocolate milk was defined as 250 ml. Other caffeine sources reported were sandwich spread, desserts, cakes and sweets containing cocoa [33]. Caffeine and nutrient calculations were performed using FoodCalc [39] and the Norwegian Food Composition Table [40]. For the purpose of this analysis, we compiled a caffeine database presenting the caffeine concentration in the main food and beverage items, depending on manner of preparation in the case of coffee, contributing to caffeine intake in the Norwegian diet (Table 1). Information about caffeine concentrations in coffee, tea and cocoa was obtained from published reports [5,41]. Caffeine concentration in soft and energy drinks was based on both figures from published reports [41] and the brewing industry. Coffee houses offered information on the content of coffee in different coffee drinks. The amount of caffeine in cocoa containing food items like chocolate was calculated based on data provided by the chocolate and food industry. The FFQ has been extensively validated in a MoBa subpopulation (n = 119) using a four-day weighed food diary and biological markers in blood and urine as reference measures [42,43]. The validation study showed that the MoBa FFQ is a valid instrument for assessing habitual diet during the first four to five months of pregnancy. The agreement between the FFQ and the food diary was particularly high for coffee (r = 0.80, 95% CI 0.72 to 0.86), and was high for tea (r = 0.53, 95% CI 0.39 to 0.65) and soft drinks (r = 0.48, 95% CI 0.33 to 0.61). Estimated caffeine intake was not evaluated at the time, but when caffeine concentrations (Table 1) were combined with consumption data for women in the validation study, high agreement was observed between the FFQ and the food dairy for total caffeine (r = 0.70, 95% CI 0.59 to 0.78). The median (IQR) caffeine intake in the validation study sample was 40 mg/day (18 to 88 mg/day) by the FFQ and 38 mg/day (10 to 99 mg/day) by the food diary. Caffeine from coffee and tea showed similar high agreement as for the beverages, while poorer agreement was seen for caffeine from chocolate (r = 0.20, 95% CI 0.02 to 0.36). No participants in the validation study had intake of caffeine from soft drinks. Food items like soft drinks, chocolate and sweets are more likely to be misreported than most other food items.

**Table 1:** Caffeine content in different food items

Food item	Caffeine (mg)/100 g
Coffee, filtered and percolated/pressed	57
Powdered instant coffee	40
Espresso	114
Cappuccino and caffè latte	21
Decaffeinated coffee	2
Caffeinated soft drinks, sugar sweetened and artificially sweetened^a^	12
Energy drinks	15^b^
Black tea	16
Chocolate milk	2
Sandwich spread containing cocoa	13
Dessert containing cocoa	3
Cake containing cocoa	4
Chocolate, medium-dark	38
Milk chocolate	15
Sweets containing cocoa	9

Caffeine content in food items included in the database used for estimating caffeine intake in the Norwegian Mother and Child Cohort Study 2002 to 2009.

^a^Caffeinated soft drinks included in the food frequency questionnaire: Coca Cola/Pepsi with sugar, Coca Cola light, Pepsi Light.

^b^Maximum caffeine content in energy drinks in Norway until 2009.

In Q1 and Q3, women reported their coffee, tea and caffeinated soft drink consumption in cups or glasses/day. These data allowed following a participant's caffeine consumption from the three main caffeine sources from the time before pregnancy until gestational week 30. Caffeine intake was entered into the analysis in mg/day, adjusted to a woman's pre-pregnancy weight and recalculated as if every woman weighed 65 kg (median pre-pregnancy weight in the study population): 65 kg × caffeine intake/pre-pregnancy weight [44].

All analyses were based on the more detailed FFQ caffeine intake data, except when the association between caffeine intake and pregnancy outcomes at different timepoints was studied using Q1 and Q3 data.

### Covariates

Information on maternal age at delivery and the baby's sex was available from the MBRN. Parity was based on both MoBa and MBRN data and categorized into number of previous pregnancies of ≥22^+0 ^weeks' duration. Marital status was defined as either married/cohabiting or not. Self-reported pre-pregnancy height and weight were used to calculate pre-pregnancy body mass index (BMI), which was categorized according to the WHO classification as underweight (<18.5 kg/m^2^), normal weight (18.5 to 24.9 kg/m^2^), overweight (25 to 29.9 kg/m^2^) and obese (≥30 kg/m^2^). Maternal education was categorized as ≤12 years, 13 to 16 years or ≥17 years. History of previous PTD (22^+0 ^to 36^+6 ^weeks of gestation), as registered in the MBRN, was taken into account as a dichotomous variable. Women reported smoking habits during pregnancy in Q1 and were categorized as non-smokers, occasional or daily smokers. Passive smoking and use of other nicotine sources were considered to be dichotomous variables. Alcohol intake from different sources was self-reported in the FFQ (glasses/day, week or month) and calculated in g/day. Persistent nausea at the time of answering the FFQ was used as a dichotomous variable. Household income was categorized as follows: participant and her partner each earning <300,000 Norwegian Krones (NOK)/year, either participant or her partner earning ≥300,000 NOK/year or participant and her partner both earning ≥300,000 NOK/year. In MoBa, more than 99% of the participants are of Caucasian ethnicity; hence ethnicity is not a relevant confounder.

### Statistics

All statistical analyses were performed using SPSS Statistics V.19.0 (SPSS, Chicago, IL, USA). Caffeine intake in relation to maternal characteristics was studied with the Kruskal-Wallis test. Associations between caffeine intake and gestational length and BW were studied with linear regression both in an unadjusted model and adjusted for the covariates mentioned above. We visually inspected residual plots to check if model assumptions were reasonably fulfilled. Odds ratios (OR) for caffeine intake and categorical outcome variables were estimated using logistic regression, both unadjusted and adjusted, as above. Statistical significance was assumed for a two-sided *P *value <0.05. Subanalyses were performed in the subgroups of non-smokers and non-coffee drinkers.

## Results

**Figure 1:**
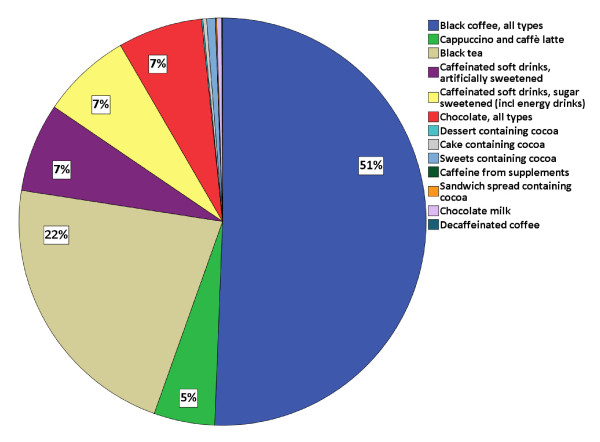
**Percentage of total caffeine intake per caffeine source**. This figure shows the percentage of total caffeine intake per caffeine source (food frequency questionnaire data), n = 59,123, in the Norwegian Mother and Child Cohort Study 2002 to 2009.

**Figure 2:**
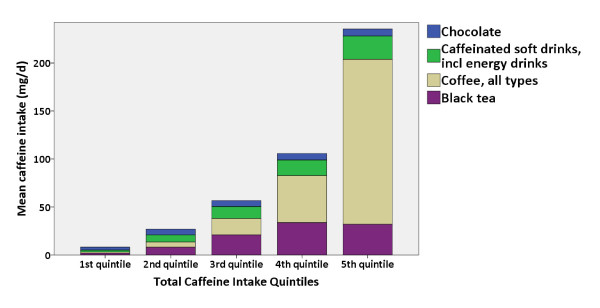
**Sources of caffeine intake according to quintiles of total caffeine intake**. This figure shows sources of caffeine intake (food frequency questionnaire data) according to quintiles of total caffeine intake, n = 59,123, in the Norwegian Mother and Child Cohort Study 2002 to 2009.

Figure 1 shows the distribution of total caffeine intake as registered in the FFQ. Coffee, black tea, soft drinks and chocolate accounted for more than 98% of daily caffeine intake but, interestingly, the dominant source differed in the low-intake and high-intake groups, with chocolate dominating in the first quintile, black tea in the second and third quintiles and coffee in the upper quintiles (Figure 2). Self-reported pre-pregnancy caffeine intake from coffee, black tea and soft drinks in Q1 and Q3 revealed a median intake of 126 mg/day (IQR 40 to 254 mg/day) for all 59,123 women, including 7,406 women who did not consume any caffeine at all. At gestational week 17 the number of non-consumers was nearly doubled (14,012 women) and the median caffeine intake had decreased to 44 mg/day (13 to 104 mg/day). At gestational week 30, the median caffeine intake had increased again to 62 mg/day (21 to 130 mg/day) and 9,792 women remained non-consumers. Caffeine intake related to maternal characteristics is presented in Table 2: older, unmarried and smoking women with higher parity, history of PTD, lower pre-pregnancy BMI, less nausea, higher energy intake and higher household income had significantly higher caffeine intake.

**Table 2:** Caffeine intake according to maternal characteristics

Characteristic	Category	n	%	Total caffeine, mg/day		Coffee caffeine, mg/day		Tea caffeine, mg/day	
				
				Median (IQR)	*P *value^a^	Median (IQR)	*P *value^a^	Median (IQR)	*P *value^a^
Total	**-**	59,123	100	57 (23 to 121)	-	7 (0 to 69)	-	5 (1 to 29)	-
Maternal age, years	<25	6,688	11	34 (15 to 81)		0 (0 to 14)		3 (0 to 15)	
	25 to 29	20,354	34	47 (19 to 102)	<10^-246^	5 (0 to 46)	<10^-246^	5 (1 to 22)	<10^-246^
	30 to 34	25,213	43	68 (27 to 134)		11 (0 to 83)		6 (1 to 34)	
	>34	6,868	12	91 (39 to 171)		30 (1 to 124)		7 (2 to 38)	
Parity	0	30,393	51	47 (19 to 103)		6 (0 to 49)		5 (1 to 23)	
	1	18,686	32	66 (26 to 133)	<10^-92^	8 (0 to 81)	<10^-92^	6 (1 to 32)	<10^-36^
	2	8,135	14	82 (31 to 163)		12 (0 to 101)		6 (1 to 35)	
	≥3	1,873	3	94 (34 to 181)		17 (0 to 138)		5 (0 to 34)	
	Missing	36	0.1	58 (22 to 142)	-	8 (0 to 98)	-	7 (0 to 30)	-
Marital status^b^	Yes	56,941	96	57 (23 to 120)	0.004	7 (0 to 69)	0.89	5 (1 to 29)	<10^-13^
	No	2,182	4	60 (21 to 146)		6 (0 to 79)		3 (0 to 20)	
Pre-pregnancy BMI, kg/m^2^	<18.5	1,835	3	69 (27 to 145)		8 (0 to 81)		7 (2 to 37)	
	18.5 to 24.9	39,834	67	62 (25 to 129)	<10^-183^	9 (0 to 79)	<10^-119^	6 (1 to 33)	<10^-237^
	25 to 29.9	12,444	21	51 (20 to 107)		5 (0 to 58)		5 (1 to 24)	
	≥30	5,01	8	39 (14 to 84)		2 (0 to 32)		2 (0 to 13)	
Maternal education, years	≤12	17,925	30	55 (21 to 128)		4 (0 to 66)		3 (0 to 19)	
	13 to 16	24,782	42	54 (22 to 114)	<10^-34^	7 (0 to 64)	<10^-124^	6 (1 to 29)	<10^-276^
	≥17	15,201	26	65 (27 to 125)		14 (0 to 79)		7 (2 to 37)	
	Missing	1,215	2	60 (22 to 124)	-	7 (0 to 69)	-	5 (0 to 28)	-
History of preterm delivery	No	57,121	97	57 (22 to 121)	<10^-6^	7 (0 to 68)	0.91	5 (1 to 29)	0.29
	Yes	1,928	3	69 (25 to 141)		7 (0 to 80)		6 (1 to 32)	
	Missing	74	0.1	47 (19 to 102)	-	12 (0 to 84)	-	5 (1 to 22)	-
Smoking habits	Never	54,136	92	54 (21 to 113)		7 (0 to 61)		6 (1 to 30)	
	Occasionally	1,569	3	109 (42 to 201)	<10^-226^	44 (0 to 163)	<10^-226^	3 (0 to 21)	<10^-101^
	Daily	3,085	5	143 (57 to 260)		70 (0 to 198)		2 (0 to 14)	
	Missing	333	1	69 (24 to 127)	-	10 (0 to 79)	-	6 (0 to 34)	-
Passive smoking	No	51,931	88	56 (22 to 118)	<10^-24^	7 (0 to 67)	0.08	6 (1 to 30)	<10^-53^
	Yes	6,082	10	66 (24 to 153)		7 (0 to 86)		3 (0 to 19)	
	Missing	1,11	2	61 (22 to 139)	-	7 (0 to 84)	-	5 (0 to 26)	-
Nicotine other than cigarettes	No	58,552	99	57 (22 to 120)	<10^-52^	7 (0 to 68)	<10^-49^	5 (1 to 29)	0.06
	Yes	571	1	138 (57 to 221)		89 (4 to 178)		4 (0 to 27)	
Alcohol consumption, units/week	No alcohol	52,47	89	53 (21 to 114)		6 (0 to 60)		5 (1 to 28)	
	<0.5	5,579	9	93 (44 to 168)	<10^-90^	39 (4 to 118)	<10^-90^	8 (2 to 37)	<10^-90^
	≥0.5	1,074	2	126 (67 to 216)		73 (13 to 173)		15 (2 to 39)	
Nausea during second trimester	No	52,567	89	59 (24 to 125)	<10^-83^	8 (0 to 74)	<10^-167^	5 (1 to 29)	<10^-10^
	Yes	6,556	11	43 (16 to 97)		0 (0 to 21)		4 (0 to 28)	
Partners with income of >300,000 NOK/year	0	16,327	28	49 (19 to 113)		4 (0 to 54)		4 (0 to 24)	
	1	24,483	41	56 (22 to 120)	<10^-72^	6 (0 to 66)	<10^-152^	5 (1 to 28)	<10^-81^
	2	16,746	28	68 (28 to 129)		14 (0 to 83)		6 (2 to 34)	
	Missing	1,567	3	57 (21 to 131)	-	5 (0 to 65)	-	4 (0 to 24)	-
Baby's sex	Male	30,049	51	58 (23 to 122)	0.13	7 (0 to 70)	0.7	5 (1 to 29)	0.62
	Female	29,074	49	57 (22 to 120)		7 (0 to 68)		5 (1 to 29)	
Quartiles of energy intake, MJ	<7.90	14,781	25	42 (15 to 96)		4 (0 to 46)		3 (0 to 21)	
	7.90 to 9.35	14,78	25	53 (21 to 112)	<10^-72^	7 (0 to 66)	<10^-75^	5 (1 to 28)	<10^-99^
	9.36 to 11.14	14,782	25	61 (25 to 126)		8 (0 to 73)		6 (1 to 31)	
	>11.14	14,78	25	78 (32 to 158)		10 (0 to 89)		6 (1 to 44)	

Caffeine intake (food frequency questionnaire data) according to maternal characteristics, n = 59,123, in the Norwegian Mother and Child Cohort Study 2002 to 2009.

^a^*P *value for being sampled from the same distribution (Kruskal-Wallis test).

^c^Marital status was defined as either married/cohabiting or not.

BMI = body mass index.

### Gestational length and spontaneous PTD

A total of 49,102 women delivered spontaneously with a median gestational length of 282 days (IQR 276 to 287 days). Gestational length as well as its residuals were left skewed, but we preferred to use the original data scale for easier interpretation.

### Caffeine intake from different sources (FFQ data)

Total caffeine intake was associated with slightly increased gestational length, that is, 5 h/100 mg/day (95% CI 3 to 8 h, *P *<10^-4^) (Table 3). However, linear regression with all different caffeine sources included in the same model revealed that only coffee caffeine was significantly associated with gestational length. When the different caffeine sources were studied individually, it emerged that the association for total caffeine intake resembled that for coffee caffeine intake (8 h/100 mg coffee caffeine/day, 95% CI 5 to 10 h, *P *<10^-7^). If all sources were studied individually without mutual adjustment, only coffee caffeine remained significantly associated with gestational length (8 h/100 mg coffee caffeine/day; 95% CI 5 to 10 h, *P *<10^-6^). As we found that coffee is the dominant source of caffeine in high-caffeine consumers, these findings could be explained by a threshold model implying that only coffee drinkers reach the threshold associated with altered gestational length. To rule out this possibility, we compared the coffee caffeine intake categorized into five groups (no intake and for the remaining subjects quartiles 0 to 8.38, 8.39 to 40.71, 40.72 to 110.52, >110.52 mg/day) finding that compared to the fifth group even group one and two had a significantly shorter gestational length (first group: regression coefficient β = -3.2, *P *= 0.04, second group: β = -4.2, *P *= 0.02). After excluding all coffee consumers from the analysis, black tea and chocolate were still not associated with gestational length while caffeinated soft drinks were associated with a 13 h decreased gestational length/100 mg additional caffeine/day (95% CI 1 to 24 h, *P *= 0.032) in the remaining 17,491 women. In the subgroup of non-coffee consumers, total caffeine intake was significantly associated with 10 h decreased gestational length/100 mg additional caffeine/day (95% CI 1 to 18 h, *P *= 0.017, adjusted models). When performing the linear regression in only non-smokers (n = 45,053), coffee caffeine was still the only caffeine source significantly associated with gestational length (total caffeine intake 7 h/100 mg caffeine/day, 95% CI 4 to 10 h, *P *<10^-5^, coffee caffeine 10 h/100 mg caffeine/day, 95% CI 7 to 13 h, *P *<10^-9^, adjusted models).

**Table 3:** Gestational length in pregnancies with spontaneous delivery and caffeine intake from different sources

Caffeine source	Unadjusted			Adjusted		
	
	β (h)	95% CI	*P *value^a^	β (h)	95% CI	*P *value^a^
Total	4	2 to 7	<10^-4^	5	3 to 8	<10^-4^
Coffee, all types	8	5 to 10	<10^-8^	8	5 to 10	<10^-7^
Caffeinated soft drinks^b^	-14	-22 to-6	<10^-3^	-8	-16 to 1	0.07
Black tea	0	-7 to 7	0.9	-1	-8 to 6	0.8
Chocolate	-42	-77 to-7	0.02	-11	-49 to 26	0.5

Gestational length in pregnancies with spontaneous delivery and caffeine intake from different sources (food frequency questionnaire (FFQ) data), linear regression for four caffeine sources as well as total caffeine intake, n = 49,102, in the Norwegian Mother and Child Cohort Study 2002 to 2009. β = change in gestational length (h) per 100 mg additional caffeine/day.

^a^*P *value, linear regression. Adjustment for maternal age, pre-pregnancy body mass index, parity, history of preterm delivery, baby's sex, nausea during second trimester, smoking habits, passive smoking, nicotine intake from other sources, alcohol consumption during pregnancy, energy intake, maternal education, marital status and household income. In the analysis of the separate caffeine sources, these were mutually adjusted (coffee, caffeinated soft drinks, black tea and chocolate).

^b^Caffeinated soft drinks included in the FFQ: Coca Cola/Pepsi with sugar, Coca Cola light, Pepsi Light.

There were 1,451 cases of spontaneous PTD in the study population (240 early spontaneous PTDs and 1,211 late spontaneous PTDs), compared to 27,498 controls, according to our strict inclusion and exclusion criteria. There was no significant association between total or coffee caffeine intake and the odds for overall, early or late spontaneous PTD (Table 4). Black tea caffeine was associated with increased risk of early spontaneous PTD (OR 1.61, 95% CI 1.10 to 2.35, *P *= 0.01, adjusted model).

**Table 4:** Odds for spontaneous preterm delivery (PTD) and caffeine intake from different sources

Caffeine source	All PTD (22^+0 ^to 36^+6 ^weeks)			Early PTD (22^+0 ^to 33^+6 ^weeks)			Late PTD (34^+0 ^to 36^+6 ^weeks)		
	
	OR^a^	95% CI	*P *value^b^	OR^a^	95% CI	*P *value^b^	OR^a^	95% CI	*P *value^b^
Total	0.98	0.92 to 1.05	0.6	0.96	0.81 to 1.14	0.7	0.99	0.92 to 1.06	0.7
Coffee, all types	0.96	0.89 to 1.04	0.3	0.82	0.66 to 1.02	0.08	0.98	0.91 to 1.07	0.7
Caffeinated soft drinks^c^	1.10	0.90 to 1.34	0.4	1.21	0.77 to 1.89	0.4	1.07	0.87 to 1.33	0.5
Black tea	0.99	0.82 to 1.20	0.9	1.61	1.10 to 2.35	0.01	0.89	0.71 to 1.11	0.3
Chocolate	1.43	0.55 to 3.67	0.5	1.08	0.10 to 12.06	0.9	1.51	0.55 to 4.15	0.4

Odds ratios for spontaneous PTD, as well as for early (n = 240) and late (n = 1,211) spontaneous PTD subgroups, and caffeine intake from different sources (food frequency questionnaire (FFQ) data), logistic regression for four caffeine sources as well as total caffeine, n = 28,949, in the Norwegian Mother and Child Cohort Study 2002 to 2009.

^a^OR: odds ratio for spontaneous PTD groups, compared to a strictly defined control group with spontaneous delivery at weeks 39^+0 ^to 40^+6^, per 100 mg additional caffeine/day.

^b^*P *value, logistic regression. Adjustment for maternal age, pre-pregnancy body mass index, parity, history of PTD, baby's sex, nausea during second trimester, smoking habits, passive smoking, nicotine intake from other sources, alcohol consumption during pregnancy, energy intake, maternal education, marital status and household income. In the analysis of the separate caffeine sources, these were mutually adjusted (coffee, caffeinated soft drinks, black tea and chocolate).

^c^Caffeinated soft drinks included in the FFQ: Coca Cola/Pepsi with sugar, Coca Cola light, Pepsi Light.

### Caffeine intake over time (Q1 and Q3 data)

At all timepoints studied, total and coffee caffeine intake was consistently associated with increased gestational length. The association was strongest for caffeine consumption reported at gestational week 17 (3 h/100 mg total caffeine/day, 95% CI 1 to 4 h; 4 h/100 mg coffee caffeine/day, 95% CI 2 to 5 h, both *P *<0.001), followed by that reported at gestational week 30 (2 h/100 mg total caffeine/day, 95% CI 0 to 3 h, *P *= 0.02; 3 h/100 mg coffee caffeine/day, 95% CI 1 to 5 h, *P *<0.001) and reported pre-pregnancy caffeine consumption (2 h/100 mg total caffeine/day, 95% CI 1 to 3 h; 2 h/100 mg coffee caffeine/day, 95% CI 1 to 3 h, both *P *<10^-6^); all adjusted models.

### Birth weight and SGA

**Figure 3:**
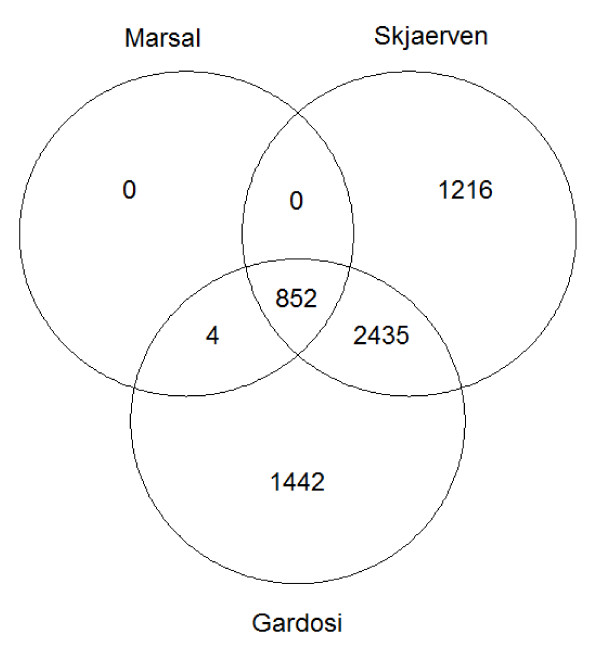
**Overlap of small for gestational age (SGA) definitions according to Marsal (ultrasound based), Skjaerven (population based) and Gardosi (customized), n = 59,123, in the Norwegian Mother and Child Cohort Study 2002 to 2009**.

The diagnosis of SGA varied considerably depending on the growth curve and SGA definition applied (Figure 3).

### Caffeine intake from different sources (FFQ data)

Total caffeine intake, as well as caffeine intake from the individual sources, was associated with lower BW (Table 5). The dependent variable in the linear regression was the difference between reported actual BW and expected BW, calculated as percentage of the expected BW. For easier understanding and interpretation, results are presented as a change in BW per 100 mg additional caffeine/day for a child with an expected BW of 3,600 g, the rounded-out median of BW in our study population (median 3,620 g). In the adjusted model, intake of an additional 100 mg total caffeine/day was associated with a 21 to 28 g BW decrease, depending on the growth curve. The opposite effect of chocolate caffeine in the Gardosi model was no longer significant after adjustment.

**Table 5:** Birth weight and caffeine intake from different sources

Caffeine source	Birth weight (Marsal)			Birth weight (Skjaerven)			Birth weight (Gardosi)		
	
	β (g)	95% CI	*P *value^a^	β (g)	95% CI	*P *value^a^	β (g)	95% CI	*P *value^a^
Unadjusted:									
Total	-28	-32 to-25	<10^-48^	-27	-31 to-23	<10^-44^	-12	-16 to-9	<10^-10^
Coffee, all types	-25	-29 to-21	<10^-30^	-22	-26 to-18	<10^-24^	-15	-19 to-11	<10^-12^
Caffeinated soft drinks^b^	-11	-24 to 1	0.08	-19	-32 to-6	0.005	-18	-30 to-5	0.006
Black tea	-53	-65 to-41	<10^-18^	-53	-65 to-42	<10^-18^	-3	-15 to 8	0.5
Chocolate	-164	-222 to-106	<10^-7^	-196	-254 to-138	<10^-10^	159	103 to 215	<10^-7^
Adjusted:									
Total	-28	-32 to-24	<10^-43^	-25	-29 to-21	<10^-34^	-21	-24 to-17	<10^-25^
Coffee, all types	-24	-28 to-19	<10^-26^	-20	-24 to-15	<10^-18^	-19	-24 to-15	<10^-19^
Caffeinated soft drinks^b^	-34	-47 to-22	<10^-7^	-38	-50 to-25	<10^-8^	-23	-35 to-11	<3 × 10^-4^
Black tea	-50	-61 to-39	<10^-17^	-48	-59 to-36	<10^-15^	-29	-40 to-18	<10^-6^
Chocolate	-129	-188 to-70	<10^-4^	-133	-192 to-73	<10^-4^	-4	-62 to 53	0.9

Birth weight (BW) and caffeine intake from different sources (food frequency questionnaire (FFQ) data), linear regression for four caffeine sources as well as total caffeine intake, n = 59,123 in the Norwegian Mother and Child Cohort Study, 2002 to 2009. β = BW decrease or increase in g per 100 mg additional caffeine/day for a baby with an expected BW of 3,600 g. Expected BW defined according to Marsal (ultrasound based), Skjaerven (population based) or Gardosi (customized).

^a^*P *value, linear regression. Adjustment for maternal age, pre-pregnancy body mass index, parity, history of preterm delivery, baby's sex, nausea during second trimester, smoking habits, passive smoking, nicotine intake from other sources, alcohol consumption during pregnancy, energy intake, maternal education, marital status, household income. In the analysis of the separate caffeine sources, these were mutually adjusted (coffee, caffeinated soft drinks, black tea and chocolate).

^b^Caffeinated soft drinks included in the FFQ: Coca Cola/Pepsi with sugar, Coca Cola light, Pepsi Light.

When studied exclusively in non-smokers (n = 54,136), these associations remained significant, again with the exception of chocolate caffeine in the Gardosi model. However, the decrease in BW was somewhat lower: Marsal 18 g (95% CI 15 to 21 g) instead of 28 g, Skjaerven 15 g (95% CI 12 to 18 g) instead of 25 g, Gardosi 12 g (95% CI 9 to 15 g) instead of 21 g per 100 mg additional total caffeine/day (all significant with *P *<10^-15^; adjusted models).

Total and coffee caffeine intake was significantly associated with higher odds for SGA, based on logistic regression in all three SGA models, both unadjusted and adjusted (Table 6). Energy drink and black tea caffeine intake were associated with a significant increase in two of the three SGA models, while there was no significant association with chocolate caffeine. The association of total caffeine intake and BW remained significant when analyses were limited to the non-smoker subgroup (n = 54,136).

**Table 6:** Odds for small for gestational age (SGA) and caffeine intake from different sources

Caffeine source	SGA (Marsal)			SGA (Skjaerven)			SGA (Gardosi)		
	
	OR^a^	95% CI	*P *value^b^	OR^a^	95% CI	*P *value^b^	OR^a^	95% CI	*P *value^b^
Unadjusted:									
Total	1.24	1.16 to 1.31	<10^-11^	1.17	1.13 to 1.20	<10^-24^	1.07	1.04 to 1.11	<10^-4^
Coffee, all types	1.21	1.13 to 1.30	<10^-7^	1.16	1.12 to 1.20	<10^-17^	1.10	1.06 to 1.13	<10^-7^
Caffeinated soft drinks^c^	1.35	1.09 to 1.67	0.007	1.27	1.14 to 1.41	<10^-5^	1.25	1.13 to 1.39	<10^-4^
Black tea	1.42	1.16 to 1.73	<10^-3^	1.20	1.09 to 1.33	<10^-3^	0.92	0.82 to 1.02	0.1
Chocolate	0.89	0.28 to 2.83	0.8	1.04	0.62 to 1.75	0.9	0.16	0.09 to 0.28	<10^-9^
Adjusted:									
Total	1.18	1.10 to 1.26	<10^-5^	1.15	1.11 to 1.19	<10^-15^	1.11	1.08 to 1.15	<10^-9^
Coffee, all types	1.14	1.06 to 1.23	<10^-3^	1.13	1.09 to 1.17	<10^-10^	1.11	1.07 to 1.16	<10^-7^
Caffeinated soft drinks^c^	1.22	0.97 to 1.53	0.08	1.29	1.16 to 1.43	<10^-5^	1.19	1.06 to 1.33	0.002
Black tea	1.50	1.22 to 1.83	<10^-4^	1.21	1.09 to 1.34	<10^-3^	1.11	0.99 to 1.23	0.06
Chocolate	1.07	0.32 to 3.54	0.9	1.06	0.61 to 1.84	0.8	0.62	0.34 to 1.12	0.1

Odds for SGA and caffeine intake from different sources (food frequency questionnaire (FFQ) data), logistic regression for four caffeine sources as well as total caffeine intake, n = 59,123 in the Norwegian Mother and Child Cohort Study, 2002 to 2009. SGA definition based on growth curves according to Marsal (ultrasound based), Skjaerven (population based) or Gardosi (customized).

^a^OR: odds ratio, compared to non-SGA, per 100 mg additional caffeine intake/day.

^b^*P *value, logistic regression. Adjustment for maternal age, pre-pregnancy body mass index, parity, history of preterm delivery, baby's sex, nausea during second trimester, smoking habits, passive smoking, nicotine intake from other sources, alcohol consumption during pregnancy, energy intake, maternal education, marital status and household income. In the analysis of the separate caffeine sources, these were mutually adjusted (coffee, caffeinated soft drinks, black tea and chocolate).

^c^Caffeinated soft drinks included in the FFQ: Coca Cola/Pepsi with sugar, Coca Cola light, Pepsi Light.

**Figure 4:**
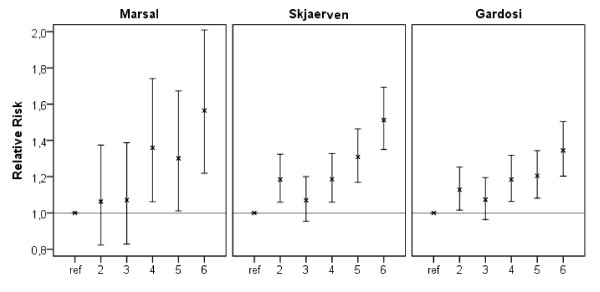
**Small for gestational age (SGA) risk depending on total caffeine intake**. Relative risk for SGA in sextiles of total caffeine intake with the lowest sextile as reference category (0 to 14.645, 14.646 to 32.093, 32.094 to 57.265, 57.266 to 96.029, 9603 to 163.806, >163.806 mg/day). SGA definitions according to Marsal (ultrasound based), Skjaerven (population based) and Gardosi (customized), n = 59,123, in the Norwegian Mother and Child Cohort Study 2002 to 2009. Adjustment for maternal age, pre-pregnancy body mass index, parity, history of preterm delivery, baby's sex, nausea during second trimester, smoking habits, passive smoking, nicotine intake from other sources, alcohol consumption during pregnancy, energy intake, maternal education, marital status and household income.

To test if there was a threshold effect, we performed the same logistic regression with sextiles of total caffeine intake (0 to 14.645, 14.646 to 32.093, 32.094 to 57.265, 57.266 to 96.029, 9603 to 163.806, >163.806 mg/day). In all three models the caffeine intake categories were associated with increasing odds ratios for SGA as compared to the lowest intake group (see Figure 4). According to FFQ data, 10.8% of all women exceeded the NNR recommendation of less than 200 mg/day caffeine intake during pregnancy and 3.3% also exceeded the WHO recommendation of less than 300 mg/day. If the odds for SGA were studied with reference to these recommendations, those 7.7% of women with a daily caffeine intake of 200 to 300 mg had significantly higher odds for SGA (1.27 to 1.62, depending on the SGA definition), in comparison with the lowest (0 to 50 mg/day) caffeine intake group. The odds of giving birth to a SGA infant were 1.62 to 1.66 in the 3.3% of women consuming >300 mg caffeine/day (Table 7).

**Table 7:** Odds for small for gestational age (SGA) and total caffeine intake according to official guidelines

Total caffeine intake groups (mg/day)	Number of individuals	SGA (Marsal)			SGA (Skjaerven)			SGA (Gardosi)		
		
		OR^a^	95% CI	*P *value^b^	OR^a^	95% CI	*P *value^b^	OR^a^	95% CI	*P *value^b^
0 to 50	27,000	1			1			1		
51 to 200	25,718	1.18	1.00 to 1.38	0.04	1.12	1.05 to 1.21	0.001	1.09	1.02 to 1.17	0.01
201 to 300	4,447	1.62	1.26 to 2.09	<10^-3^	1.44	1.28 to 1.62	<10^-8^	1.27	1.12 to 1.44	<10^-3^
>300	1,958	1.62	1.15 to 2.29	0.006	1.66	1.41 to 1.96	<10^-8^	1.62	1.36 to 1.92	<10^-7^

Odds ratios for SGA and total caffeine intake (food frequency questionnaire (FFQ) data), according to official Nordic Nutrition Recommendations (<200 mg caffeine/day) and World Health Organization (<300 mg caffeine/day) guidelines, logistic regression for n = 59,123 in the Norwegian Mother and Child Cohort Study, 2002 to 2009. SGA defined according to Marsal (ultrasound based), Skjaerven (population based) or Gardosi (customized).

^a^OR: odds ratio, compared to lowest caffeine intake group (0 to 50 mg/day).

^b^*P *value, logistic regression. Adjustment for maternal age, pre-pregnancy body mass index, parity, history of preterm delivery, baby's sex, nausea during second trimester, smoking habits, passive smoking, nicotine intake from other sources, alcohol consumption during pregnancy, energy intake, maternal education, marital status and household income.

### Caffeine intake over time (Q1 and Q3 data)

Total and coffee caffeine intake at all timepoints studied was significantly associated with decreased BW for all applied SGA models. The association with caffeine intake from black tea was the strongest with the Skjaerven and Marsal models. Tea caffeine was not significantly associated with BW if defined according to Gardosi, though. For caffeine from coffee and soft drinks, intake reported at gestational week 17 had the strongest impact on BW (Table 8).

**Table 8:** Birth weight and maternal caffeine intake at different timepoints before and during pregnancy

Model^a^	Caffeine source	Birth weight (Marsal)			Birth weight (Skjaerven)			Birth weight (Gardosi)		
		
		β (g) 95% CI		*P *value	β (g) 95% CI		*P *value	β (g) 95% CI		*P *value
Pre-pregnancy:										
I	Total	-3	-5 to-2	<10^-6^	-2	-4 to-1	<10^-3^	-1	-3 to 0	0.04
	Coffee, all types	-3	-4 to-2	<10^-4^	-2	-3 to 0	0.02	-1	-3 to 0	0.07
	Caffeinated soft drinks^b^	-3	-7 to 1	0.1	-4	-8 to 0	0.06	-1	-5 to 3	0.6
	Black tea	-16	-23 to-9	<10^-5^	-16	-23 to-8	<10^-4^	-6	-13 to 1	0.1
II	Total	1	-1 to 3	0.3	2	0 to 3	0.03	2	1 to 4	0.003
	Coffee, all types	1	-1 to 3	0.2	2	0 to 4	0.02	3	1 to 4	<3 × 10^-3^
	Caffeinated soft drinks^b^	4	-3 to 10	0.3	3	-3 to 10	0.3	5	-1 to 11	0.1
	Black tea	-12	-21 to-2	0.02	-13	-22 to-3	0.01	-3	-12 to 7	0.6
17th week of gestation:										
I	Total	-9	-12 to-7	<10^-17^	-8	-10 to-6	<10^-13^	-7	-9 to-5	<10^-10^
	Coffee, all types	-9	-11 to-6	<10^-11^	-7	-10 to-5	<10^-7^	-7	-10 to-5	<10^-8^
	Caffeinated soft drinks^b^	-9	-15 to-4	<2 × 10^-3^	-10	-15 to-4	<10^-3^	-6	-11 to-1	0.03
	Black tea	-14	-22 to-6	<10^-3^	-12	-20 to-4	<5 × 10^-3^	-6	-14 to 2	0.13
II	Total	-8	-11 to-6	<10^-8^	-8	-10 to-5	<10^-8^	-7	-10 to-5	<10^-8^
	Coffee, all types	-8	-11 to-4	<10^-5^	-8	-11 to-4	<10^-5^	-7	-10 to-4	<10^-4^
	Caffeinated soft drinks^b^	-13	-22 to-5	<3 × 10^-3^	-13	-22 to-4	<4 × 10^-3^	-12	-20 to-3	<7 × 10^-3^
	Black tea	-1	-12 to 9	0.8	2	-9 to 13	0.8	-3	-14 to 7	0.5
30th week of gestation:										
I	Total	-8	-10 to-6	<10^-13^	-7	-9 to-5	<10^-10^	-6	-8 to-4	<10^-7^
	Coffee, all types	-9	-11 to-6	<10^-10^	-7	-9 to-4	<10^-7^	-7	-10 to-5	<10^-8^
	Caffeinated soft drinks^b^	-2	-6 to 3	0.5	-3	-7 to 2	0.3	0	-5 to 4	0.9
	Black tea	-18	-27 to-10	<10^-4^	-18	-27 to-9	<10^-4^	-8	-16 to 1	0.08
II	Total	-5	-7 to-3	<10^-4^	-5	-7 to-3	<10^-4^	-4	-7 to-2	<10^-3^
	Coffee, all types	-6	-9 to-3	<10^-3^	-5	-9 to-2	<2 × 10^-3^	-6	-9 to-3	<10^-4^
	Caffeinated soft drinks^b^	-1	-6 to 5	0.8	-1	-6 to 4	0.7	0	-5 to 5	0.9
	Black tea	-14	-24 to-4	<5 × 10^-3^	-15	-25 to-5	<3 × 10^-3^	-6	-16 to 3	0.2

Birth weight (BW) and maternal caffeine intake reported at different timepoints before and during pregnancy (data from Q1 and Q3), linear regression for three caffeine sources as well as total caffeine intake, n = 59,123 in the Norwegian Mother and Child Cohort Study, 2002 to 2009. β = BW gain (in g) per 100 mg additional caffeine/day for a baby with an expected BW of 3,600 g.

^a^*P *value, linear regression adjusted as follows. Model I: maternal age, pre-pregnancy body mass index, parity, history of preterm delivery, baby's sex, nausea during second trimester, smoking habits, passive smoking, nicotine intake from other sources, alcohol consumption during pregnancy, energy intake, maternal education, marital status, household income. In the analysis of the separate caffeine sources, these were mutually adjusted (coffee, caffeinated soft drinks, black tea and chocolate). Model II: as model I, as well as for caffeine intake from different sources as reported at the other studied timepoints.

^b^Caffeinated soft drinks included in Q1 and Q3: Coca Cola/Pepsi with sugar, Coca Cola light, Pepsi Light.

## Discussion

Caffeine from coffee, but not from other sources, was associated with slightly increased gestational length. Total caffeine and caffeine from all different sources studied was associated with decreased BW. When discussing these results, the caffeine intake pattern in this Norwegian subpopulation must be kept in mind: the dominant caffeine source varied with increasing total caffeine intake, from chocolate in the low consumption group, to black tea in the medium consumption group, and coffee in the high consumption group. Thus, findings attributed to increasing total caffeine intake might be due to a changing distribution of caffeine sources. These results emphasize what Peck *et al.* and the CARE Study Group pointed out: if the aim of an epidemiologic study is to assess the effect of caffeine, it is not correct to study only coffee caffeine [20,45].

Many, but not all, women decreased their caffeine consumption considerably during the first trimester but increased it again during the second trimester, a motive for repeated caffeine intake measurements during pregnancy in studies examining exposure in relation to pregnancy outcome [20,45].

### Gestational length and spontaneous PTD

We found that total and coffee caffeine intake was associated with a highly significant increase of gestational length by 5 and 8 hours/100 mg respectively. The corresponding association with total caffeine intake was, conversely, 10 hours decreased gestational length, though only marginally significant, when coffee drinkers were excluded from the model. Additionally, we ruled out a threshold effect, as even the groups with the lowest coffee caffeine intake were significantly associated with gestational length. Our results do not support the hypothesis that caffeine intake influences the risk for spontaneous PTD. The only marginally significant finding was black tea caffeine being associated with higher PTD odds, in the relatively small subgroup of early spontaneous PTD. We therefore conclude that the association of total caffeine intake with gestational length is not related to caffeine but to coffee intake. This study was not designed to disclose the reason for this statistical association; one possible explanation is that there might be some other substance, present in coffee but not in the other caffeine sources, that influences gestational length. Human parturition is depending on a physiological inflammatory reaction leading to cervical ripening and increased uterus tonus [46]. Melanoidins that are generated from coffee bean components during the roasting process are, for example, known to have antimicrobial and anti-inflammatory effects [47] and thus might influence the timing of parturition. People in Scandinavia who do not drink coffee constitute a definite minority and those with a very low caffeine intake are probably a special group in many other ways. Drinking coffee, but not consuming other caffeine sources, might be associated with gestational length by some lifestyle factor.

The most important confounder on the behavioral level is smoking. Smokers are known to have higher coffee consumption [20]; furthermore, smoking is an established risk factor for spontaneous PTD. According to our results, however, coffee drinking and smoking have opposing effects on gestational length. The association with coffee intake remained significant after adjusting for smoking and excluding all smokers from the analysis, strongly suggesting an association between coffee consumption and gestational length that is independent of smoking behavior.

There was no major difference regarding the association for coffee consumption during different periods of pregnancy, suggesting either a rather continuous effect of coffee drinking on gestational length or confounding of coffee consumption with some other factor, as opposed to coffee consumption at a specific timepoint affecting some crucial step of pregnancy development.

In summary, we seem to have identified an association for coffee rather than caffeine, which must be kept in mind when discussing our findings in the context of earlier publications. To the best of our knowledge, this study is the first to separately study the associations between gestational length and caffeine from coffee, on the one hand, and caffeine from other sources, on the other. In comparison, most observational studies have not found any associations between caffeine or coffee and gestational length [48-53] and Maslova *et al.* found no significant association with overall PTD risk in their meta-analysis [31]. There may be several reasons for the fact that we found an association for coffee drinking, but not for caffeine per se. We used self-reported caffeine intake instead of measuring caffeine metabolites [49,50]. Caffeine from several sources was assessed, rather than caffeine intake from a single source, usually coffee, as in many studies [30,53-55]. For some populations using only caffeine from coffee would implicate that a major, if not the major, part of total caffeine intake was not considered at all, for example, in a UK study black tea contributed 62% to daily caffeine intake while coffee and cola drinks accounted for about 12% to 14% each [45]. PTD is a heterogeneous group of pregnancy outcomes with heterogeneous etiology [32]. In contrast to the studies mentioned above, we defined a clear spontaneous PTD phenotype by excluding all iatrogenic deliveries as well as all medical or obstetric complications. The etiologies of early and late spontaneous PTD differ, which has not been acknowledged in many other studies [20]. Mikkelsen *et al.* found an increased risk of early, but not late, overall PTD related to coffee intake of more than 2 cups/day in the Danish Birth Cohort [30], while Haugen *et al.* failed to confirm these results in an earlier version of MoBa [55]. In this study, in which only spontaneous PTD in uncomplicated pregnancies was investigated, black tea caffeine intake was significantly associated with increased risk for early spontaneous PTD while significant associations were not found for other caffeine sources, indicating that this association was not caused by caffeine. The association with black tea caffeine was only marginally significant and there was no significant association between black tea caffeine and gestational length so that these results should be interpreted with caution.

In addition to the above-mentioned paper by Mikkelsen *et al.*, Klonoff-Cohen *et al.* reported decreased gestational length related to caffeine consumption of >50 mg/day, compared to 0 to 2 mg/day, in a sample of 39 pregnancies [29]. To our knowledge, this study of 49,102 pregnancies with spontaneous delivery is the biggest and most detailed observational study so far on the association between caffeine intake and gestational length, particularly spontaneous PTD. Our data do not support the hypothesis that caffeine intake or coffee consumption decrease gestational length or increase the risk for spontaneous PTD. Although we did find a significant association, a change in gestational length of several hours probably lacks clinical implications.

### Birth weight and SGA

We found significant associations between caffeine intake and SGA and decreased BW. These associations were strengthened by concordant results for different caffeine sources, comparable overall findings regardless of the growth standard and SGA definition applied, remaining significance after adjustment, biological gradient, stability over period of pregnancy, consistency with other studies and biological plausibility. Caffeine is metabolized more slowly during pregnancy, crosses the placental barrier [7] and increases maternal levels of 3'5'-cyclic monophosphate and epinephrine [56], causing uteroplacental vasoconstriction and decreased intervillous placental blood flow, which could restrict fetal growth [48,54]. Another hypothesis, postulated by Weathersbee *et al.*, is that caffeine inhibits phosphodiesterase, leading to an increase in cellular cyclic adenosine monophosphate, which may interfere with fetal growth [57].

Smoking is a difficult confounder when studying effects of caffeine consumption [20]. Both smoking and caffeine intake are associated with lower BW. However, the association between caffeine intake and BW remained highly significant after adjustment for smoking and after analysis in the non-smoker subgroup, suggesting an independent association with caffeine consumption.

Caffeine intake reported at gestational week 17 was most strongly associated with BW. This could be explained by reverse causality, according to Lawson's hypothesis, that is, the placenta is comparatively smaller in pregnancies complicated by SGA than in healthy pregnancies, thus producing less hormones and evoking fewer pregnancy symptoms so that these women might maintain a higher caffeine intake [20,58]. However, remaining significance after controlling for nausea and the finding of a significant association for pre-pregnancy caffeine intake as well contradicts reverse causality as only explanation.

While some earlier studies found no significant association between caffeine consumption and BW [48,53,54,59], most publications are consistent with our findings in MoBa [49,50,60-63]. Especially the data from some of the largest observational studies published so far, are consistent with our findings, moreover with comparable effect size of a decrease in BW by 60 to 70 g for >200 mg/day [45] or 28 g for 100 mg/day caffeine consumption [50].

In this study population, more than 10% exceeded the NNR recommended maximum intake of 200 mg/day; this subgroup had 20% to 60% higher odds ratios for SGA. Although SGA babies are generally known to be at higher risk for both neonatal morbidity and mortality [24], this might not be true for babies born SGA due to maternal caffeine consumption. Hernandez-Diaz found that babies born SGA due to maternal smoking might have lower mortality than other SGA babies with more severe causes for being born SGA, such as congenital malformations [64]. However, as our results confirm earlier findings [45] that the increase in SGA risk can already be found in women following the current recommendations by Norwegian Authorities, further studies are needed to establish the impact of caffeine on neonatal morbidity and mortality. We could not find a threshold for the association of caffeine consumption and SGA risk. Until there is clarity if there is a causal association between caffeine intake and increased risk for SGA, women might be advised to reduce their caffeine consumption as much as possible during pregnancy.

### Limitation and strengths

To the best of our knowledge, with its sample size of 59,123 pregnancies, this is the largest study performed so far on the association between caffeine intake and pregnancy outcome. The MoBa participation rate is 38.5%, and demographic comparison with the MBRN in 2002 showed that single women and women aged <25 are underrepresented in MoBa. Regarding SGA (4.6% in MoBa and 5.1% in the MBRN) and PTD (7.2% in MoBa and 7.7% in the MBRN), the differences are minor and the subgroup composition is similar to that in the total population, with spontaneous PTD accounting for 42% of all PTD [28]. Additionally, a recent study found no bias in eight selected exposure-outcome associations [65].

Due to the large study sample, there were 1,411 cases defined as spontaneous PTD and 852 (Marsal), 4,503 (Skjaerven) or 4,733 (Gardosi) cases of SGA in the study population. Estimation of gestational length by second-trimester ultrasound and clear definition of a spontaneous PTD phenotype are additional strengths of this study [20,24,32]. Different standard growth curves were applied and this study is one of the first caffeine effect studies using customized growth curves at all [20,45]. The overall results for all three models indicate an adverse effect of caffeine consumption on SGA risk, strengthening the association found.

All dietary assessment methods have limitations, and so does the self-reported caffeine intake in this study. The mean caffeine concentrations in seven main categories of food and drinks were used for the exposure calculations, while large variations may actually exist within each category [41]. The caffeine contributed by soft drinks is likely to be underestimated as Coca Cola and Pepsi (including their diet versions) were the only soft drinks distinguished from other soft drinks in the FFQ. However, some other soft drinks also contain caffeine, for example, Urge, a citrus flavored soft drink produced by Coca Cola Norway. However, this soft drink comprised a rather small share of the market.

Relying exclusively on self-reported data without a biological marker to confirm the accuracy of estimated caffeine exposure is a weakness. For the present study we evaluated the agreement between caffeine intake estimated by the FFQ and a food diary. The estimated caffeine intake did not differ between the methods, and a high correlation was observed (r = 0.70, 95% CI 0.59 to 0.78). Furthermore, the MoBa FFQ has been extensively validated in a MoBa subpopulation using the four-day weighed food diary and several biomarkers as reference measures [42,43]. The agreement between the FFQ and the food diary was particularly high for coffee and tea, which are the main sources of caffeine in this study. Coffee intake according to both the FFQ and the food diary correlated with serum β-carotene (0.31 and 0.36, respectively), which can be explained by interplay between antioxidants in coffee with β-carotene, as also reported by Svilaas *et al.*[66]. Likewise, tea intake according to both the FFQ and the food diary correlated with kaempferol, a flavonoid found in tea (r = 0.41 and 0.50 for the FFQ and food diary, respectively [42]). Similar, but slightly weaker correlations were observed for estimated caffeine contributed by coffee and tea. A Bland-Altman plot for the differences in caffeine intake between the FFQ and the food diary is available as Additional file 1.

There are further strengths related to the caffeine intake assessment: the prospective design ensured that women's responses were not influenced by their knowledge of pregnancy outcome. Caffeine intake assessment from different sources, as well as different coffee preparations being taken into account, are also clear strengths of this study [20]. As the FFQ covers the first four to five months of pregnancy, when many women change their dietary habits due to nausea, some women may have had difficulties reporting the frequency and amount of caffeine consumption for the whole period correctly. Coffee and black tea intake varies less and is easier to recall than intake of most other food groups. The associations with caffeine intake based on the FFQ were corroborated by caffeine intake estimates based on reported consumption of caffeine-containing drinks in the other two questionnaires (Q1 and Q3). As the relevant window of susceptibility for caffeine effects is not yet known [20], caffeine consumption assessment at different timepoints is a further strength of this study.

There is always a possibility of residual confounding in observational studies, but we reduced this possibility by controlling for a number of relevant factors, including history of PTD, nausea and smoking. Our smoking variable has been shown to be a valid marker for tobacco use when tested against plasma cotinine concentration [67].

## Conclusions

Coffee intake is associated with slightly increased gestational length but does not affect the odds for spontaneous PTD. It is not caffeine, however, that is the cause of this association. Whether it is some other substance present in coffee, but not in other caffeine sources, or whether coffee drinking is associated, on a behavioral level, with some factor influencing gestational length remains to be further investigated.

Caffeine intake is associated with decreased BW and increased odds for SGA. These associations are strengthened by concordant results for different caffeine sources, comparable overall findings regardless of the growth curve or definition of SGA, remaining significance after adjustment, stability over period of pregnancy, consistency with other studies and biological plausibility. These SGA babies might be at higher risk for both short-term and long-term morbidity. As the risk for SGA increases even if pregnant women follow official recommendations in Norway of a maximum caffeine intake of 200 mg/day, this association should be further investigated and recommendations might have to be re-evaluated.

## Abbreviations

BMI: body mass index; BW: birth weight; CI: confidence interval; FFQ: food frequency questionnaire; IQR: interquartile range; MoBa: Norwegian Mother and Child Cohort Study; MBRN: Medical Birth Registry of Norway; NNR: Nordic Nutrition Recommendations; NOK: Norwegian Krone (currency); OR: odds ratio; PTD: preterm delivery; SGA: small for gestational age; WHO: World Health Organization.

## Competing interests

The authors declare that they have no competing interests.

## Authors' contributions

VS, EE, JB, SN, MH, HMM, JA, BJ and A-LB planned the study. VS, RM and BJ identified preterm and term deliveries. VS, JB, SN, JG and BJ identified SGA deliveries. EE, MH, HMM, JA and A-LB calculated caffeine intake from the FFQ. VS, JB and SN analyzed the data. All authors contributed to interpretation of results and writing the paper. All authors have read and approved the manuscript for publication.

## Authors' information

VS and BJ are obstetricians at the Department of Obstetrics and Gynaecology, Sahlgrenska University Hospital/Östra, Gothenburg, Sweden, a department with more than 10,000 deliveries/year and a specialized ward for high-risk pregnancies. MH designed the MoBa FFQ. HMM and JA contributed to the development and implementation of the MoBa FFQ. A-LB validated the MoBa FFQ. EE collected information on caffeine content in Norwegian foods and constructed the caffeine database. MH, HMM, and A-LB have extensive experience of epidemiological studies involving data emanating from the MoBa FFQ. JB has a background in biochemistry. SN and JG have a broad experience in biostatistics and epidemiology.

## Pre-publication history

The pre-publication history for this paper can be accessed here:

http://www.biomedcentral.com/1741-7015/11/42/prepub

## Supplementary Material

Additional file 1 **Bland-Altman plot for the difference in caffeine intake between the food frequency questionnaire (FFQ) and a four-day weighed food diary in 119 women in the validation study**. Bland-Altman plot of the differences in caffeine intake between the FFQ and the food diary measurements (bias) against the mean caffeine intake by the two methods showing that the mean difference was small and not biased towards any of the methods. The median (IQR) caffeine intake in the validation study sample was 40 mg/day (18 to 88 mg/day) by the FFQ and 38 mg/day (10 to 99 mg/day) by the food diary. Spearman correlation was 0.70 (95% CI 0.59 to 0.78). Click here for file
