# Supplementary figures and images for: Maternal caffeine intake during pregnancy is associated with birth weight but not with gestational length: results from a large prospective observational cohort study
Source: BMC Med. 2013 Feb 19;11:42. doi: 10.1186/1741-7015-11-42 (PMC3606471; doi:10.1186/1741-7015-11-42)

## Slide 1
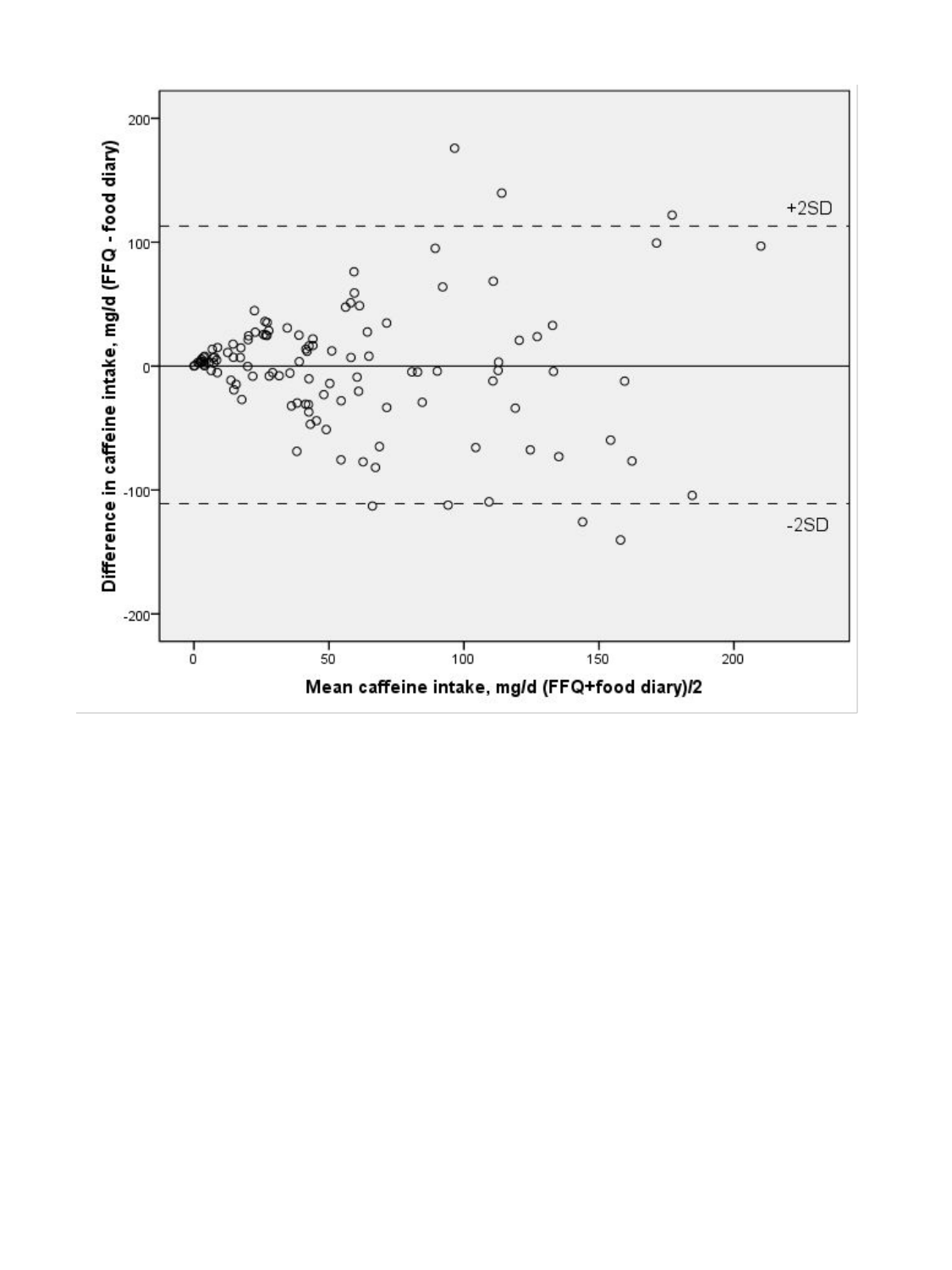

Supplement: Additional file 1 — Bland-Altman plot for the difference in caffeine intake between the food frequency questionnaire (FFQ) and a four-day weighed food diary in 119 women in the validation study. Bland-Altman plot of the differences in caffeine intake between the FFQ and the food diary measurements (bias) against the mean caffeine intake by the two methods showing that the mean difference was small and not biased towards any of the methods. The median (IQR) caffeine intake in the validation study sample was 40 mg/day (18 to 88 mg/day) by the FFQ and 38 mg/day (10 to 99 mg/day) by the food diary. Spearman correlation was 0.70 (95% CI 0.59 to 0.78). [file 1741-7015-11-42-S1.PPTX]
